# In vitro suppression of immune responses using monocyte-derived tolerogenic dendritic cells from patients with primary Sjögren's syndrome

**DOI:** 10.1186/ar4294

**Published:** 2013-09-09

**Authors:** Roman Volchenkov, Johan G Brun, Roland Jonsson, Silke Appel

**Affiliations:** 1 Broegelmann Research Laboratory, Department of Clinical Science, University of Bergen, Jonas Lies vei 65, 5021 Bergen, Norway; 2 Department of Rheumatology, Haukeland University Hospital, Jonas Lies vei 65, 5021 Bergen, Norway; 3 Section for Rheumatology, Department of Clinical Science, University of Bergen, Jonas Lies vei 65, 5021 Bergen, Norway

**Keywords:** Sjögren's syndrome, dendritic cells, immunotherapy, Ro/La-specific T cell suppression

## Abstract

**Introduction:**

Therapeutic vaccination with antigen-specific tolerogenic dendritic cells (tolDC) might become a future option of individualized therapy for patients with autoimmune diseases. In this study, we tested the possibility of generating monocyte-derived tolDC from patients with primary Sjögren's syndrome (pSS). We analyzed phenotype, cytokine production and ability to suppress Ro/La-specific immune responses.

**Methods:**

Monocyte-derived tolDC from patients with pSS were generated in the presence of dexamethasone, vitamin D3 and lipopolysaccharide (DexVD3 DC). The phenotype was analyzed by flow cytometry and the cytokine profile was investigated using a 25-plex Luminex assay and ELISA. The capacity to both stimulate Ro/La-specific T cells and suppress this response was evaluated by autologous mixed lymphocyte reaction (MLR).

**Results:**

DC generated from patients with pSS had a similar phenotype and cytokine profile to those from healthy controls. DexVD3 DC from pSS patients induced little antigen-specific T cell proliferation, but DexVD3 DC-primed lymphocytes successfully suppressed Ro/La-specific T cell responses.

**Conclusions:**

DexVD3 DC presenting Ro/La antigens might be a promising new therapeutic option for patients with pSS.

## Introduction

Primary Sjögren's syndrome (pSS) is a chronic autoimmune disease, characterized by mononuclear cell infiltrations, preferentially in salivary and lacrimal glands that lead to xerostomia and keratoconjunctivitis sicca, respectively. Similar to other autoimmune diseases, the understanding of the pathogenesis of pSS and its etiology is far from complete [1]. Most of the patients are females (9:1 female-to-male ratio) and presence of autoantibodies against SSA (Ro52 and Ro60) and SSB (La) antigens is observed in 50 to 60% and 30 to 40% of patients with pSS, respectively [2]. The current therapies mostly alleviate the symptoms of sicca and focuses on extraglandular manifestations (if present) [3]. The results of clinical trials with biological treatments showed minimal or no effect in patients with pSS [4,5]. Therefore, there is an ongoing need for individualized patient treatment. Therapeutic vaccination with monocyte-derived tolerogenic dendritic cells (tolDC) might be a future treatment option.

Dendritic cells are unique cells of the immune system that are bridging innate and adaptive immunity [6]. They are responsible for the initiation of immune responses and are involved in regulation of central and peripheral tolerance [7,8]. For more than a decade, tolDC have been studied in animal models of autoimmune diseases, and they have been shown to both prevent the disease and treat already established autoimmune conditions [9]. Several protocols for the generation of tolDC have been developed including modification through pharmacological treatment of monocytes with dexamethasone, rapamycin and nuclear factor kappa B (NF-κB) inhibitors [10]. One of the described protocols for the generation of tolDC includes combined treatment of monocytes with glucocorticoid dexamethasone, 1α,25-dihydroxyvitamin D3 and lipopolysaccharide (LPS) [11]. This protocol performs better than other tolDC treatments *in vitro *[12] and has been show to be efficient in a mouse model of rheumatoid arthritis (RA) [13].

Despite the success of tolDC treatments in mice, the application of the method in humans is not widely established yet. So far, successful generation of tolDC and induction of antigen-specific T cell hyporesponsiveness was reported only for RA and multiple sclerosis (MS) patients [14,15]. In our study we addressed the feasibility of generating functional tolDC from patients with pSS as it was previously reported that the monocytes from these patients are functionally impaired [16].

## Materials and methods

### Patient material

Blood samples from pSS patients fulfilling the American-European classification criteria for pSS [17] (*n *= 12) were collected at the Department of Rheumatology, Haukeland University Hospital, Bergen, Norway. The control group consisted of five gender- and age-matched healthy blood donors from the Blood Bank at Haukeland University Hospital, Bergen, Norway. The characteristics of patients are given in Table 1.

**Table 1:** Clinical data on patients used in the study.

Cohort characteristics			
		Sjögren's syndrome	Healthy controls
Number		*n *= 12	*n *= 5
Age in years (mean +/- SD)		50 +/-13	45 +/-7
Range		33-71	34-52
Female:male		12:0	5:0
Time since diagnosis in years (mean +/- SD)		8.5 +/- 4.1	
Clinical features			
			
Anti-Ro		10/12	
Anti-La		5/12	
Both anti-Ro and anti-La		5/12	
No anti-Ro and anti-La		2/12	
Rheumatoid factor		4/12	
Anti-ribonucleoprotein		1/12	
			
Focus score			
	FS:0	3/12	
	FS:1	2/12	
	FS:2	5/12	
	FS:3	0/12	
	FS:4	2/12	
	FS:5	0/12	
			
Treatment			
	Antimalarial	4/12	
	Methotrexate	1/12	
			
Extraglandular manifestations		8/12	
	Hypothyriodism	2/12	
	Polyarthritis	2/12	
	Exanthema	2/12	
	Polyneuropathy	1/12	
	Pulmonary embolism	1/12	

The study was approved by the Ethics Committee at the University of Bergen (Approval number 242.06) and all subjects (both patients and healthy blood donors) signed the informed consent according to the Declaration of Helsinki.

### Generation of dendritic cells

Dendritic cells (DC) were generated from monocytes isolated from fresh blood collected into heparin tubes from pSS patients and healthy blood donors as described previously [12]. The autologous peripheral blood mononuclear cells (PBMC) depleted for monocytes (nonadherent cells, NAC) were cryopreserved in X-VIVO20 medium with 10% dimethyl sulfoxide (DMSO; Sigma-Aldrich, St. Louis, MO, USA) and stored at -80°C until further use. DexVD3 DC were generated by addition of 1 μM dexamethasone (Sigma-Aldrich, St. Louis, MO, USA) at day 3 and dexamethasone plus 0.1 nM 1α,25-dihydroxyvitamin D3 (Enzo Life Sciences, Laussen, Switzerland) at day 6. Since DMSO was used as a solvent for all compounds the equivalent amount of DMSO was added to the control populations (DMSO DC) on days 3 and 6. On day 6, the cells were either incubated with 1 μg/ml tuberculin purified protein derivative (PPD, Statens Serum Institut, Copenhagen, Denmark) or a mixture of recombinant Ro52, Ro60 and La protein (1 μg/ml each, all from Arotec Diagnostics, Wellington, New Zealand). DexVD3 DC and half of the DMSO DC were stimulated with LPS (100 ng/ml, Sigma-Aldrich, St. Louis, MO, USA) at the time of antigen supplement. Cells were harvested 24 h after the stimulation.

### Flowcytometry

Immunostaining was performed as described previously [12]. Briefly, after 5 min incubation with Fc receptor block (Miltenyi, Bergisch Gladbach, Germany) cells were stained with a titrated amount of antibodies for 10 min in the dark at room temperature before being washed and immediately analyzed on a LSRFortessa cytometer (BD Biosciences, Heidelberg, Germany). All subsequent analyses were done with FlowJo software (Tree Star, Ashland, OR, USA). One percent false-positive events were accepted in the negative controls. The antibodies used for DC phenotyping were CD1a-PE (HI149), CD14-FITC (18D11) from ImmunoTools (Friesoythe, Germany); HLA-DR-Horizon V500 (G46-6), CD83-PE-CF594 (HB15e) from BD Biosciences (Heidelberg, Germany); CD38-PerCP-Cy5.5 (HIT2), CD86-Alexa Fluor 647 (IT2.2), CD80-Brilliant Violet 605 (2D10), CD40-PE-Cy7 (5C3); CCR7-Brilliant Violet 421 (G043H7), all from BioLegend (San Diego, CA, USA).

### Cytokine determination

Cell-free supernatants from DC cultures were stored in aliquots at -20°C. Production of cytokines, chemokines and growth factors was analyzed with a Cytokine Human Magnetic 25-plex panel assay (Life Technologies, Glasgow, UK) on a Luminex 100 System (Luminex Corporation, Austin, TX, USA) according to the manufacturer's instructions. Levels of B-cell activating factor (BAFF) in supernatants were measured with the Quantikine Human BAFF/BLyS ELISA from R&D Systems (Minneapolis, MN, USA).

### T cell stimulatory capacity

To analyze the capacity of the generated DC populations to induce antigen-specific T cell responses, an autologous mixed lymphocyte reaction (MLR) was utilized. The autologous PBMC depleted for monocytes (nonadherent cells, NAC) were thawed and allowed to rest overnight before being labeled with CellTrace Violet Cell Proliferation Kit (Invitrogen, Carlsbad, CA, USA) according to the manufacturer's recommendations. A total of 200,000 CellTrace Violet-labeled NAC were then co-cultured with 40,000 autologous DC previously incubated with antigen (PPD or Ro/La). After 5 days the cells were harvested, stained for CD4 (CD4-APC, MEM-241, from ImmunoTools, Friesoythe, Germany) and proliferation was analyzed on an LSRFortessa flow cytometer. For the induction of Ro/La-specific T cells, only patients positive for Ro or La were used.

### Suppression experiments

To analyze the suppressive capacity of lymphocytes primed with the different DC populations (immature DMSO DC, LPS-stimulated DMSO DC, LPS-stimulated DexVD3 DC), autologous NAC of Ro/La autoantibody-positive patients were thawed and allowed to rest overnight before priming with tolDC (1:6 DC to NAC ratio) for 5 days. Then the nonadherent lymphocytes were harvested, washed and rested for another 5 days. After the rest, these cells were harvested, washed, counted and labeled using the CellTrace Violet Cell Proliferation Kit (Invitrogen, Carlsbad, CA, USA) according to the manufacturer's instructions. Mature DMSO DC previously pulsed with Ro and La antigens and autologous naive NAC (responder cells) were thawed and allowed to rest overnight. Then the responder cells were labeled with CFDA-SE (Invitrogen, Carlsbad, CA, USA) according to the manufacturer's instructions to prevent convergence with DC-primed NAC. Responder cells were incubated with DC-primed cells and in the presence of mature DMSO DC (ratio 2:1:0.2). After the co-culture for 5 days the cells were harvested and proliferation was analyzed on an LSRFortessa flow cytometer.

All co-culture experiments and resting phases were carried out in X-VIVO20 medium supplemented with IL-2 (50 U/ml; ImmunoTools, Friesoythe; Germany).

### Statistical analysis

Mann-Whitney *U *test was used for group-wise statistical analyses. Significance was set at *P *< 0.05. All statistical calculations were done with Prism 5 (GraphPad Software, Inc., La Jolla, CA, USA).

## Results

### Monocyte-derived DC from patients with pSS have a similar phenotype as DC from healthy controls

**Figure 1:**
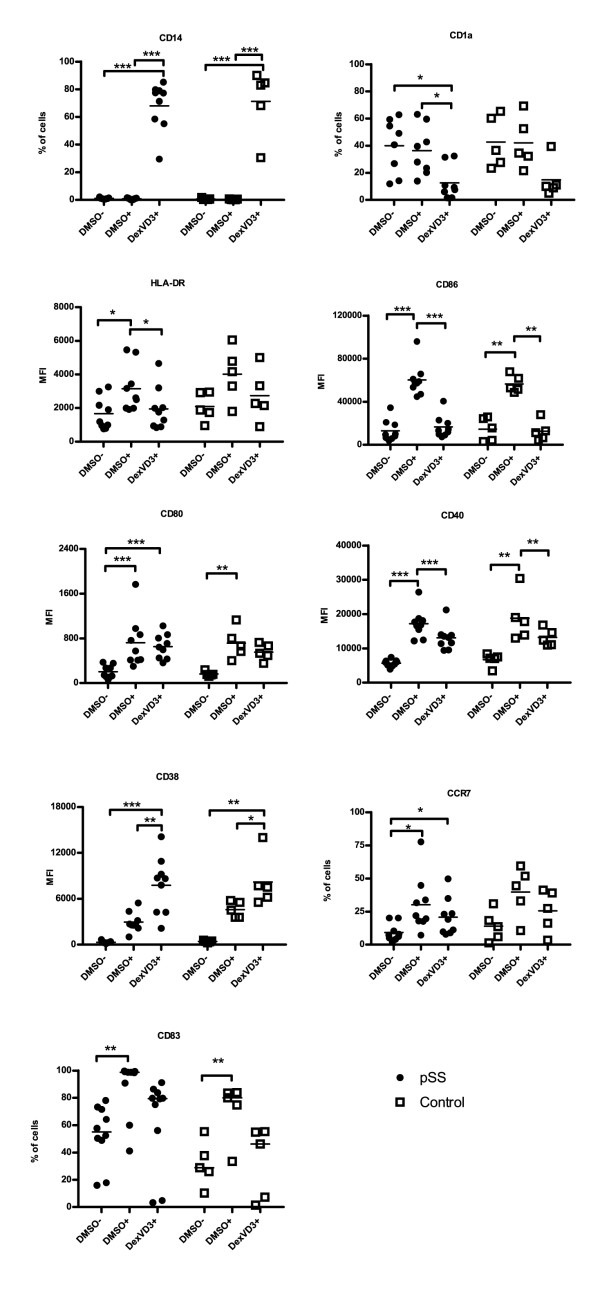
**Monocyte-derived tolDC from patients with pSS have a similar phenotype to tolDC from healthy controls**. The percentage of positive cells or median fluorescence intensity (MFI) is shown. MFI was used when all DC populations showed more than 90 percent positivity. Each symbol represents an independent sample, *n *= 9 for DC from patients with pSS (filled circles) and *n *= 5 for DC from healthy controls (open squares). Differences between the expression of surface markers are marked with the following: *P ≤ 0.05; **P ≤ 0.01; ***P ≤ 0.001. The bars indicate median. DMSO-, immature DC; DMSO+, LPS-stimulated DC; DexVD3+, LPS-stimulated DC generated in the presence of dexamethasone and 1alpha,25-dihydroxyvitamin D3; pSS, primary Sjögren's syndrome; tolDC, tolerogenic dendritic cells.

First, we investigated the phenotype of the three DC populations generated from patients with pSS in comparison to cells from age- and gender-matched healthy controls (Figure 1; see Figure S1 in Additional file 1). Immature DMSO DC in both groups were characterized by low levels of MHC class II molecules, and lower levels of co-stimulatory molecules CD80, CD86, CD40, CD83, and migration markers CD38 and CCR7. Stimulation of DMSO DC with LPS upregulated the expression of those molecules (Figure 1). Both immature and mature DMSO DC expressed relatively high levels of the DC marker CD1a, and no monocyte-macrophage marker CD14 was detected on their surface. DexVD3 DC from patients with pSS and controls had a semi-mature macrophage-like phenotype with low CD1a and high CD14 expression, low MHC class II, CD40, CD80, CD83, CD86, and CCR7. CD38 was expressed significantly higher on DexVD3 DC in comparison to mature DMSO DC in both pSS and controls (*P *= 0.005 and *P *= 0.01, respectively). The results were similar for patients with and without anti-rheumatic treatment.

### DexVD3 DC generated from patients with pSS are efficient IL-10 producers

**Figure 2:**
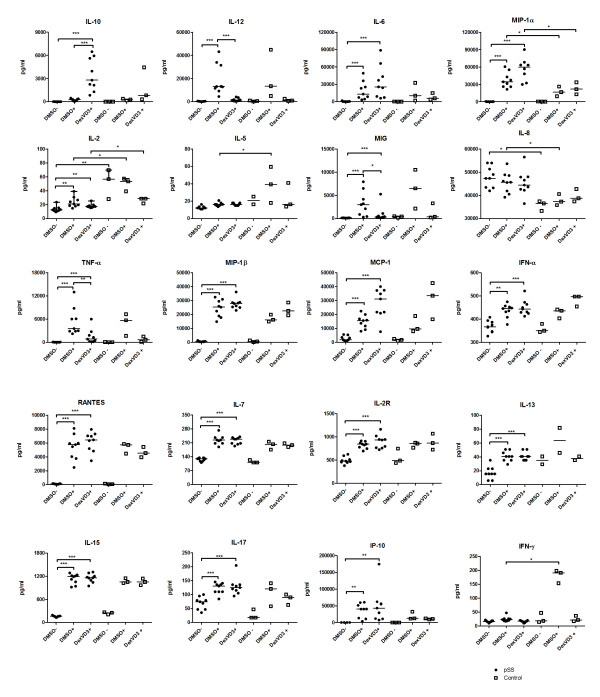
**Cytokine profile of DexVD3 DC generated from patients with pSS**. The cell-free supernatants were collected when harvesting the DC populations and stored in aliquots at -20°C until analysis. The amount of cytokines and chemokines secreted were analyzed using a 25-plex bead assay and the concentrations are given in pg/ml. Each symbol represents an independent sample, *n *= 9 for supernatants from DC from patients with pSS (filled circles) and *n *= 3 for supernatants from DC from healthy controls (open squares). GM-CSF and IL-4 were part of the added cytokines and therefore not included in the analysis. Cytokines and chemokines without significant differences between the groups (IL-1β, IL-1RA and Eotaxin) are not shown. The bars show the median and the differences between the secreted amounts of cytokines are marked with the following: *P ≤ 0.05; **P ≤ 0.01; ***P ≤ 0.001. DMSO-, immature DC; DMSO+, LPS-stimulated DC; DexVD3+, LPS-stimulated DC generated in the presence of dexamethasone and 1alpha,25-dihydroxyvitamin D3; GM-CSF, granulocyte-macrophage colony-stimulating factor; pSS, primary Sjögren's syndrome.

Next, the supernatants from DC populations generated from patients with pSS and healthy controls were analyzed using a 25-plex Luminex assay (Figure 2). LPS-stimulated DMSO DC from patients with pSS produced significantly higher amounts of macrophage inflammatory protein-1α (MIP-1α; CCL3) and IL-8, and significantly lower quantities of IFN-γ and IL-5 compared to mature DMSO DC from healthy controls. DexVD3 DC from patients with pSS produced significantly higher amounts of the anti-inflammatory cytokine IL-10 in comparison to both immature and mature DMSO DC. Those DC also secreted significantly lower amounts of proinflammatory cytokines IL-12 and TNF-α as well as chemokine monokine induced by gamma interferon (MIG; CXCL9) in comparison to mature DMSO DC. Both mature DMSO DC and DexVD3 DC generated from pSS patients produced significantly higher quantities of cytokines and chemokines IL-6, -7, -13, -15, -17, interferon gamma-induced protein 10 (IP-10; CXCL10), IL-2R, MIP-1α, MIP-1β (CCL4), monocyte chemotactic protein-1 (MCP-1; CCL2), IFN-α, RANTES (CCL5; Figure 2) in comparison to immature DMSO DC generated from pSS patients. In supernatants of DC generated from healthy controls similar trends were observed, however, the differences were not significant. DexVD3 DC from patients with pSS produced significantly higher amounts of MIP-1α in comparison to DexVD3 DC generated from healthy controls. The only cytokine that was produced in higher quantities by all DC populations generated from healthy controls compared to pSS patients was IL-2.

None of the generated DC populations produced any BAFF (data not shown).

Anti-rheumatic treatment did not have an effect on cytokine and chemokine production of the monocyte-derived DC populations.

### DexVD3 DC-primed NAC from patients with pSS suppress antigen-specific T cell proliferation

**Figure 3:**
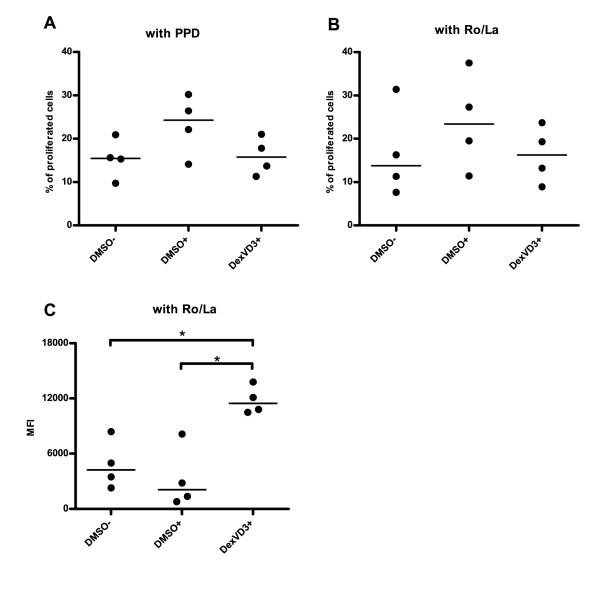
**DexVD3 DC primed lymphocytes from patients with pSS suppress antigen-specific T cell proliferation**. **(A) **First, DC from a group of patients with pSS (*n *= 4) were pulsed with 1 μg/ml PPD and co-cultured with CellTrace Violet-labeled autologous NAC (1:5 ratio) and the percentage of proliferated cells was analyzed. **(B**) Next, this experiment was repeated with another group of patients (*n *= 4) using a mixture of Ro52, Ro60 and La (1 μg/ml each). **(C) **Naïve NAC (responder cells labeled with CFSE) were co-cultured with different DC-primed NAC (effector cells labeled with CellTrace Violet) in the presence of Ag-loaded mature DMSO DC (ratio 2:1:0.2) for 5 days. CellTrace Violet-positive cells were gated out and the level of proliferation of CFSE-labeled responder cells was evaluated as the reduction of MFI. The DC population that was used to stimulate the effector cells is shown on the x-axis. DMSO-, immature DC;, DMSO+, LPS-stimulated DC; DexVD3+, LPS-stimulated DC generated in the presence of dexamethasone and 1alpha,25-dihydroxyvitamin D3; NAC, nonadherent cells; PPD, purified protein derivative; pSS, primary Sjögren's syndrome. Each dot represents one individual experiment, bars indicate median, *n *= 4 (same patients as in Figure 3B), *P ≤ 0.05.

Next, we determined the immunostimulatory capacity of the three DC populations generated from patients with pSS using autologous NAC and PPD as a recall antigen. NAC were labeled with CellTrace Violet and its dilution was measured after co-culture with PPD-primed DC (for gating strategy see Figure S2 in Additional file 2). In line with their phenotype and cytokine production, immature DMSO DC and DexVD3 DC induced less proliferation (median 15.3% and 13.7%, respectively) when compared to LPS-stimulated DMSO DC (median 22.1%) (Figure 3A). However, the difference did not reach statistical significance (*P *> 0.05).

Then, we evaluated if this was also the case when using pSS-related autoantigens Ro52, Ro60 and La48. First, we measured the proliferation levels of autologous NAC after co-culture with the three DC populations. Similar to the experiments with PPD as an antigen, LPS-stimulated DMSO DC induced the highest proliferation rate (up to 37.5%, median 23.4%). DexVD3 DC performed at similar efficiency as immature DMSO DC (median 16.5% and 13.8%, respectively, *P *> 0.05) (Figure 3B).

After the co-culture and removal of the DC, the NAC were rested for 5 days. The cells were then used as effector cells in order to test their ability to suppress naive T cell proliferation upon stimulation with mature DMSO DC loaded with Ro52, Ro60 and La48. Addition of NAC previously primed with DexVD3 DC to the co-culture reaction resulted in significantly reduced proliferation of responder cells when compared to both immature and mature DMSO DC (Figure 3C; for gating strategy see Figure S3 in Additional file 3). The results were not affected by the medication of some of the patients as similar results were obtained for all patients included.

**Figure 4:**
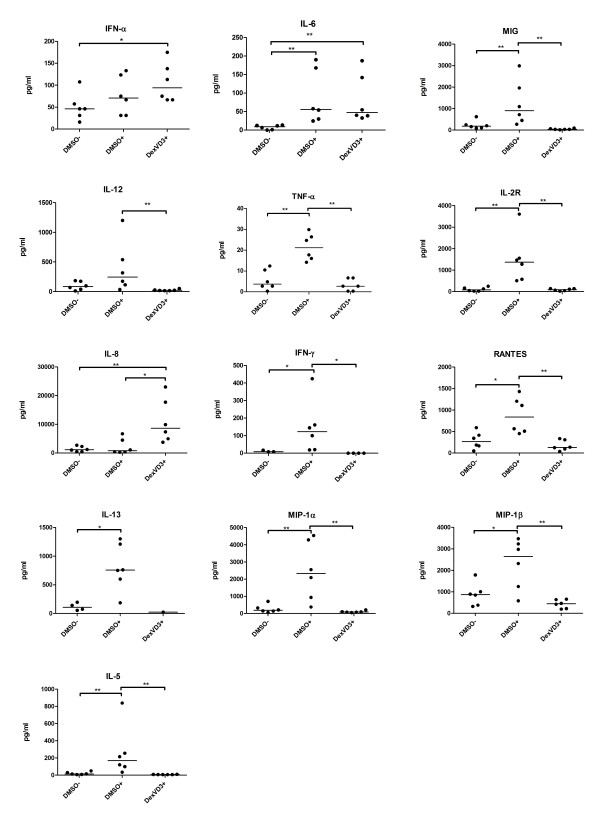
**NAC previously primed with DexVD3 DC secrete IL-8 during the rest phase**. Cytokine and chemokine profiling was performed on culture supernatants from NAC previously primed with various DC populations generated from pSS patients. The cell-free culture supernatants were collected after co-culture of DC and NAC and stored in aliquots at -20°C until analysis. The amount of cytokines/chemokines secreted was analyzed using a 25-plex bead assay and the concentrations are given in pg/ml. The bars show the median, *n *= 6. The differences between the secreted amounts of cytokines are marked with the following: *P ≤ 0.05; **P ≤ 0.01. Cytokines and chemokines that showed results below the detection limit (IL-10, Eotaxin, IL-17, GM-CSF, IL-1β, IL-4) and those that where the differences were not significant are not shown. The DC population that was used to stimulate NAC is shown on the x-axis. See Figure 1 for definitions. GM-CSF, granulocyte-macrophage colony-stimulating factor; NAC, nonadherent cells.

The supernatants from both, resting NAC and suppression co-cultures, were analyzed using a 25-plex Luminex assay. During the resting phase, the NAC previously primed with mature DMSO DC secreted significantly higher amounts of TNF-α, IFN-γ, RANTES, MIP-1α, MIP-1β, IL-2R, and -5, when compared to NAC previously primed with immature DMSO DC and DexVD3 DC (Figure 4). The IL-12 production by NAC primed with mature DMSO DC was significantly higher when compared to DexVD3 DC, but not to immature DMSO DC. Both NAC primed with mature DMSO DC and DexVD3 DC produced higher amounts of IL-6 in comparison to NAC primed with immature DMSO DC. Furthermore, NAC primed with DexVD3 DC produced significantly higher amounts of IFN-α (vs. NAC primed with immature DMSO DC) and IL-8 (vs. both NAC primed with immature and mature DMSO DC).

**Figure 5:**
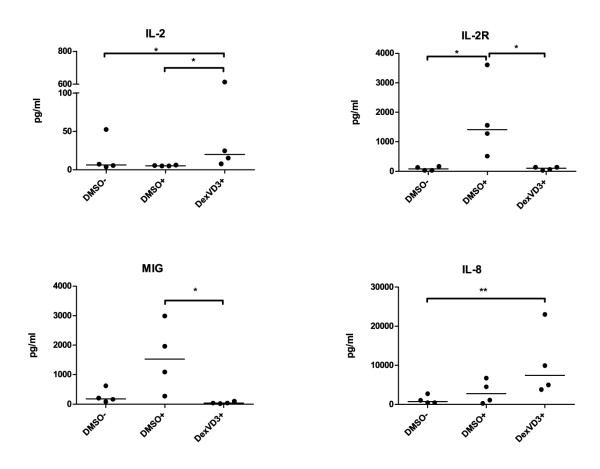
**IL-2 and IL-8 are produced in larger quantities in suppression experiments with effector cells primed by DexVD3 DC**. The supernatants were collected before the FACS analysis of proliferation of the responder cells and stored in aliquots at -20°C until analysis. The amount of cytokines/chemokines secreted were analyzed using a 25-plex bead assay and the concentrations are given in pg/ml. Cytokines and chemokines that showed results below the detection limit (IL-1β, IL-4, Eotaxin, GM-CSF, IL-17), and those that where the differences were not significant, are not shown. The bars indicate the median, *n *= 4. The differences between the secreted amounts of cytokines are marked with the following: *P ≤ 0.05.

In the supernatants from the suppression co-cultures with effector cells primed by DexVD3 DC, we detected significantly higher levels of IL-8 (vs. primed by immature DMSO DC; Figure 5) and IL-2 (vs. both primed by immature and mature DMSO DC IL-2R). In addition to that, in co-cultures with effector cells primed by mature DMSO DC higher levels of IL-2R and MIG were detected (Figure 5). The medication of some of the patients did not influence the results.

## Discussion

Our study demonstrates the successful generation of monocyte-derived tolDC from patients with pSS using the previously established robust protocol that relies on the combined effect of dexamethasone, vitamin D3 and Toll-like receptor 4 (TLR4) ligand LPS [11,12]. DexVD3 DC generated from patients with pSS had a typical semi-mature phenotype similar to those generated from healthy controls.

Interestingly, we observed a high expression of CD38 on DexVD3 DC, both from patients with pSS and controls. Under normal conditions, CD38 is highly expressed on monocytes and when monocytes turn into immature DC the expression of CD38 decreases [18]. During the maturation of DC it is upregulated to the same extent as on monocytes [18]. The significantly increased expression of CD38 on DexVD3 DC might favor good migratory capacity of DexVD3 DC, as CD38 is associated with migration and survival of human DC [19], and might be an additional indicator of their semi-mature phenotype. Indeed, in an experimental model of RA, DexVD3 DC efficiently migrated from the site of injection to lymph nodes and secondary lymphoid organs [13]. CD38 expression might therefore be useful as a quality control marker for tolDC [14]. Moreover, high CD38 expression on DexVD3 DC might be involved in the induction of type 1 regulatory T cells (Tr1 cells) by these DC [13] as CD38 is a ligand for CD31, a molecule that is required for T cell tolerance [20].

Similar to our previous study with DC generated from healthy blood donors, the DexVD3 DC generated from pSS patients produced the highest amounts of IL-10 and little IL-12 [12]. In contrast to our previous observation on DC generated from healthy blood donors, the DexVD3 DC from pSS patients secreted increased amounts of IL-6, -7, -13, -15, -17, IP-10, IL-2R, MIP-1α, MIP-1β, MCP-1, IFN-α, TNF-α and RANTES compared to immature DMSO DC. Furthermore, the quantities of these cytokines were even higher than those produced by mature DMSO DC from pSS patients, although the differences were not significant. The observed increased production of proinflammatory cytokines and chemokines in DC from pSS patients might be due to systemic inflammation and activation of compensatory mechanisms. However, these DC were still able to induce antigen-specific suppressor cells, indicating a dominant role for IL-10.

It has previously been reported that monocytes from pSS patients produced high levels of IL-6 and BAFF [16]. We were, therefore, interested to see if this would be carried over to the monocyte-derived DC from pSS patients. Although we observed significant differences in IL-6 production between different groups of DC generated from pSS patients, there were no differences in IL-6 production between DC generated from pSS patients and healthy controls. It was reported previously that IL-6 is important in T helper 17 (Th17) cell differentiation, as IL-6 influences the balance between regulatory T cells (Treg) and Th17 cells in favor of inflammation [21-24]. However, in most of these studies, the transcription factor FoxP3 was used to define Tregs, while numerous studies have demonstrated that this is not a suitable marker for cells with regulatory functions in humans [25-27]. In addition, the vaccination with DexVD3 DC in collagen-induced arthritis was associated with a reduction of Th17 cells [13]. Therefore, the increase of IL-6 production by DexVD3 DC observed in our study might be compensated for by their tolerogenic effects. Moreover, no BAFF was detected in supernatants from monocyte-derived DC. Therefore, we believe that tolDC generated from monocytes of pSS patients do not have the same functional impairment as monocytes. However, the previously mentioned study by Yoshimoto *et al*. used an in-house ELISA for the detection of BAFF with a higher sensitivity in comparison to commercially available ELISAs [16].

One of the important considerations for immunotherapy with tolDC is the ability of tolDC to overcome the immunogenic DC that initiate immune response toward self-antigens in the patients. In our study, DexVD3 DC-primed NAC were able to suppress stimulation of naïve T cells in the presence of fully matured DC loaded with pSS-related autoantigens Ro52, Ro60 and La. We have previously shown that DexVD3 DC induce both type 1 T regulatory cells (Tr1) and B regulatory cells (Breg) [12]. It seems, therefore, likely that the observed suppression described here is most likely mediated by Tr1 and Breg cells [12,13]. The Tr1 cells utilize several mechanisms to perform their regulatory function. These include production of inhibitory cytokines, release of proteases (Granzyme A) and cell-to-cell contact [28]. The latter mechanisms might be used by DexVD3 DC-induced Tr1 cells, as we observed no increase in anti-inflammatory cytokines in the supernatants from neither resting NAC nor suppression co-cultures. However, our results are limited by the small sample size in our suppression experiments as it was not possible to include all patients in these experiments. The number of PBMC we were able to isolate from the limited amounts of peripheral blood from each patient restricted the number of experiments we could perform with cells from each patient.

Interestingly, resting NAC primed by DexVD3 DC secreted considerably higher amounts of IL-8 in our experiments. A similar increase of IL-8 production was observed during the suppression experiments with NAC primed by DexVD3 DC. IL-8 is a proinflammatory cytokine that is involved in T cell chemotaxis [29,30]. However, it is also a signature cytokine for the Treg cell line HOZOT-4, established from human umbilical cord blood [31,32] and can be produced by FoxP3+ Treg [33]. Since we previously showed that the DexVD3 DC induce Tr1 cells and not FoxP3+ Treg [12], it would be interesting to see whether IL-8 is involved in regulatory function of Tr1.

Although promising, our results are limited by the fact that autoantibodies against Ro and La are not present in all patients and different patients might have different epitopes to which their immune systems react. Indeed, in patients without Ro and La autoantibodies, we did not observe a significant suppression of proliferation by DexVD3 DC-primed effector cells (data not shown). In RA patients, it was proposed to use synovial fluid as an autologous source of autoantigens [34], but for pSS such a source of disease-specific autologous autoantigens has not yet been found. Therefore, GMP-compatible recombinant proteins for large-scale pilot studies or preclinical trials have to be produced. The time of application of a tolDC vaccine to pSS patients is another complicated issue. Usually, it takes several years to establish a diagnosis and often at this time the damage to salivary glands is irreversible [1]. On the other hand, although some studies have found associations between the degree of destruction and loss of function [35-37], at the patient level there is not necessarily such a correlation.

## Conclusions

In summary, this is the first study demonstrating the successful generation of Ro/La-loaded tolDC from patients with pSS able to efficiently induce antigen-specific suppressive immune reactions. As the described protocol can easily be adjusted to be GMP compatible [14], we believe that tolDC might be a potentially favourable treatment option for patients with pSS with known antibody specificity.

## Abbreviations

BAFF: B-cell activating factor; Breg: regulatory B cells; GM-CSF: granulocyte-macrophage colony-stimulating factor; IL: interleukin; INF: interferon; IP-10: interferon gamma-induced protein 10; LPS: lipopolysaccharide; MCP-1: monocyte chemotactic protein-1; MFI: median fluorescence intensity; MIG: monokine induced by gamma interferon; MIP-1α: macrophage inflammatory protein-1α; MS: multiple sclerosis; NAC: nonadherent cells; PPD: purified protein derivative; pSS: primary Sjögren's syndrome; RA: rheumatoid arthritis; Th: T helper; TNF: tumor necrosis factor; tolDC: tolerogenic dendritic cells; Tr1: type 1 regulatory T cells.

## Competing interests

The authors declare that they have no competing interests.

This work was supported by the Broegelmann Foundation and the Bergen Research Foundation.

## Authors' contributions

RV participated in study design, performed experiments and statistical analysis and drafted the manuscript. SA conceived the study, participated in its design and helped to draft the manuscript. RJ coordinated the study and helped to draft the manuscript. JGB provided patients' sample material and helped to draft the manuscript. All authors read and approved the final manuscript.

## Supplementary Material

Additional file 1 **Figure S1. DexVD3 DC have a semi-mature macrophage-like phenotype**. The DC were gated based on forward and side scatter dot plots and the expression of surface markers was analyzed. Each histogram shows the negative control (red), immature DMSO DC (blue), mature DMSO DC (orange) and DexVD3 DC (green). Click here for file

Additional file 2 **Figure S2. Gating strategy for the analysis of T cell proliferation after priming with various DC groups**. The top left dot plot depicts cells in a live gate based on forward scatter and side scatter, the top right dot plot depicts gating on CD4^+ ^and CD8^+ ^T cells. The bottom left histogram represents proliferation of CD8^+ ^T cells and the bottom right histogram shows the proliferation of CD4^+ ^T cells. Click here for file

Additional file 3 **Figure S3. Gating strategy for the analysis of the suppressive capacity of lymphocytes primed with various tolDC populations**. Naïve responder cells were labeled with CFSE and tolDC-primed NAC were labeled with CellTrace Violet. This allowed exclusive gating on CellTrace Violet-negative cells and further analysis of proliferation of CFSE-labeled responder cells. The top left dot plot depicts cells in a live gate based on forward scatter and side scatter, the top right histogram depicts gating on CellTrace Violet-negative cells. The bottom histogram shows proliferation of CFSE-labeled CellTrace Violet-negative responder cells after co-culture with differently primed NAC. MFI values of proliferated responder cells and tolDC populations that were used to stimulate effector cells are shown in the histograms legend. MFI, median fluorescence intensity; NAC, nonadherent cells; tolDC, tolerogenic dendritic cells. Click here for file

## References

[B1] Jonsson R Vogelsang P Volchenkov R Espinosa A Wahren-Herlenius M Appel S The complexity of Sjogren's syndrome: novel aspects on pathogenesis Immunol Lett 2011 15 1 9 21777618 10.1016/j.imlet.2011.06.007

[B2] Volchenkov R Jonsson R Appel S Anti-Ro and anti-La autoantibody profiling in Norwegian patients with primary Sjogren's syndrome using luciferase immunoprecipitation systems (LIPS) Scand J Rheumatol 2012 15 314 315 22804348 10.3109/03009742.2012.670863

[B3] Brito-Zeron P Siso-Almirall A Bove A Kostov BA Ramos-Casals M Primary Sjogren syndrome: an update on current pharmacotherapy options and future directions Expert Opin Pharmacother 2013 15 279 289 23346917 10.1517/14656566.2013.767333

[B4] Mariette X Ravaud P Steinfeld S Baron G Goetz J Hachulla E Combe B Puechal X Pennec Y Sauvezie B Perdriger A Hayem G Janin A Sibilia J Inefficacy of infliximab in primary Sjogren's syndrome: results of the randomized, controlled Trial of Remicade in Primary Sjogren's Syndrome (TRIPSS) Arthritis Rheum 2004 15 1270 1276 15077311 10.1002/art.20146

[B5] Devauchelle-Pensec V Mariette X Jousse-Joulin S Berthelot J-M Perdriger A Tolerance and efficacy of rituximab in primary Sjogren's syndrome: final results of a randomized controlled trial Arthritis Rheum 2012 15 S1079

[B6] Banchereau J Steinman RM Dendritic cells and the control of immunity Nature 1998 15 245 252 9521319 10.1038/32588

[B7] Steinman RM Turley S Mellman I Inaba K The induction of tolerance by dendritic cells that have captured apoptotic cells J Exp Med 2000 15 411 416 10662786 10.1084/jem.191.3.411 PMC2195815

[B8] Liu YJ A unified theory of central tolerance in the thymus Trends Immunol 2006 15 215 221 16580260 10.1016/j.it.2006.03.004

[B9] Thomson AW Robbins PD Tolerogenic dendritic cells for autoimmune disease and transplantation Ann Rheum Dis 2008 15 Suppl 3 iii90 96 19022823 10.1136/ard.2008.099176

[B10] Morelli AE Thomson AW Tolerogenic dendritic cells and the quest for transplant tolerance Nat Rev Immunol 2007 15 610 621 17627284 10.1038/nri2132

[B11] Anderson AE Swan DJ Sayers BL Harry RA Patterson AM von Delwig A Robinson JH Isaacs JD Hilkens CM LPS activation is required for migratory activity and antigen presentation by tolerogenic dendritic cells J Leukoc Biol 2009 15 243 250 18971286 10.1189/jlb.0608374 PMC2700018

[B12] Volchenkov R Karlsen M Jonsson R Appel S Type 1 regulatory T cells and regulatory B cells induced by tolerogenic dendritic cells Scand J Immunol 2013 15 246 254 23442246 10.1111/sji.12039

[B13] Stoop JN Harry RA von Delwig A Isaacs JD Robinson JH Hilkens CM Therapeutic effect of tolerogenic dendritic cells in established collagen-induced arthritis is associated with a reduction in Th17 responses Arthritis Rheum 2010 15 3656 3665 20862679 10.1002/art.27756

[B14] Harry RA Anderson AE Isaacs JD Hilkens CM Generation and characterisation of therapeutic tolerogenic dendritic cells for rheumatoid arthritis Ann Rheum Dis 2010 15 2042 2050 20551157 10.1136/ard.2009.126383 PMC3002758

[B15] Raiotach-Regue D Grau-Lopez L Naranjo-Gomez M Ramo-Tello C Pujol-Borrell R Martinez-Caceres E Borras FE Stable antigen-specific T-cell hyporesponsiveness induced by tolerogenic dendritic cells from multiple sclerosis patients Eur J Immunol 2012 15 771 782 22488365 10.1002/eji.201141835

[B16] Yoshimoto K Tanaka M Kojima M Setoyama Y Kameda H Suzuki K Tsuzaka K Ogawa Y Tsubota K Abe T Takeuchi T Regulatory mechanisms for the production of BAFF and IL-6 are impaired in monocytes of patients of primary Sjogren's syndrome Arthritis Res Ther 2011 15 R170 22018243 10.1186/ar3493 PMC3308105

[B17] Vitali C Bombardieri S Jonsson R Moutsopoulos HM Alexander EL Carsons SE Daniels TE Fox PC Fox RI Kassan SS Pillemer SR Talal N Weisman MH European Study Group on Classification Criteria for Sjogren's S Classification criteria for Sjogren's syndrome: a revised version of the European criteria proposed by the American-European Consensus Group Ann Rheum Dis 2002 15 554 558 12006334 10.1136/ard.61.6.554 PMC1754137

[B18] Fedele G Frasca L Palazzo R Ferrero E Malavasi F Ausiello CM CD38 is expressed on human mature monocyte-derived dendritic cells and is functionally involved in CD83 expression and IL-12 induction Eur J Immunol 2004 15 1342 1350 15114667 10.1002/eji.200324728

[B19] Frasca L Fedele G Deaglio S Capuano C Palazzo R Vaisitti T Malavasi F Ausiello CM CD38 orchestrates migration, survival, and Th1 immune response of human mature dendritic cells Blood 2006 15 2392 2399 16293598 10.1182/blood-2005-07-2913

[B20] Ma L Mauro C Cornish GH Chai JG Coe D Fu H Patton D Okkenhaug K Franzoso G Dyson J Nourshargh S Marelli-Berg FM Ig gene-like molecule CD31 plays a nonredundant role in the regulation of T-cell immunity and tolerance Proc Natl Acad Sci USA 2010 15 19461 19466 20978210 10.1073/pnas.1011748107 PMC2984185

[B21] Samson M Audia S Janikashvili N Ciudad M Trad M Fraszczak J Ornetti P Maillefert JF Miossec P Bonnotte B Brief report: inhibition of interleukin-6 function corrects Th17/Treg cell imbalance in patients with rheumatoid arthritis Arthritis Rheum 2012 15 2499 2503 22488116 10.1002/art.34477

[B22] Ryba-Stanislawowska M Skrzypkowska M Mysliwska J Mysliwiec M The serum IL-6 profile and Treg/Th17 peripheral cell populations in patients with type 1 diabetes Mediators Inflamm 2013 15 205284 23533301 10.1155/2013/205284 PMC3595664

[B23] Lee DG Woo JW Kwok SK Cho ML Park SH MRP8 promotes Th17 differentiation via upregulation of IL-6 production by fibroblast-like synoviocytes in rheumatoid arthritis Exp Mol Med 2013 15 e20 23619188 10.1038/emm.2013.39 PMC3641402

[B24] Tanaka T Kishimoto T Targeting interleukin-6: all the way to treat autoimmune and inflammatory diseases Int J Biol Sci 2012 15 1227 1236 23136551 10.7150/ijbs.4666 PMC3491446

[B25] Morgan ME van Bilsen JH Bakker AM Heemskerk B Schilham MW Hartgers FC Elferink BG van der Zanden L de Vries RR Huizinga TW Ottenhoff TH Toes RE Expression of FOXP3 mRNA is not confined to CD4+CD25+ T regulatory cells in humans Hum Immunol 2005 15 13 20 15620457 10.1016/j.humimm.2004.05.016

[B26] Gavin MA Torgerson TR Houston E DeRoos P Ho WY Stray-Pedersen A Ocheltree EL Greenberg PD Ochs HD Rudensky AY Single-cell analysis of normal and FOXP3-mutant human T cells: FOXP3 expression without regulatory T cell development Proc Natl Acad Sci USA 2006 15 6659 6664 16617117 10.1073/pnas.0509484103 PMC1458937

[B27] Wang J Ioan-Facsinay A van der Voort EI Huizinga TW Toes RE Transient expression of FOXP3 in human activated nonregulatory CD4+ T cells Eur J Immunol 2007 15 129 138 17154262 10.1002/eji.200636435

[B28] Gregori S Goudy KS Roncarolo MG The cellular and molecular mechanisms of immuno-suppression by human type 1 regulatory T cells Front Immunol 2012 15 30 22566914 10.3389/fimmu.2012.00030 PMC3342353

[B29] Wechsler AS Gordon MC Dendorfer U LeClair KP Induction of IL-8 expression in T cells uses the CD28 costimulatory pathway J Immunol 1994 15 2515 2523 8077662

[B30] Gesser B Lund M Lohse N Vestergaad C Matsushima K Sindet-Pedersen S Jensen SL Thestrup-Pedersen K Larsen CG IL-8 induces T cell chemotaxis, suppresses IL-4, and up-regulates IL-8 production by CD4+ T cells J Leukoc Biol 1996 15 407 411 8604020 10.1002/jlb.59.3.407

[B31] Sugimoto A Suzuki M Otani T Okochi A Takeuchi M Yamasaki F Nakamura S Kibata M HOZOTs, novel human regulatory T-cell lines, exhibit helper or suppressor activities depending on dendritic cell or anti-CD3 stimulation Exp Hematol 2009 15 1454 1463 19819295 10.1016/j.exphem.2009.10.002

[B32] Harashima A Toraya T Okochi A Yamamoto M Suzuki M Otani T Inoue T Tsuji-Takayama K Sugimoto A Takeuchi M Yamasaki F Nakamura S Kibata M Interleukin-8 and RANTES are signature cytokines made by HOZOT, a new type of regulatory T cells Mol Immunol 2009 15 3310 3319 19699525 10.1016/j.molimm.2009.07.023

[B33] Himmel ME Crome SQ Ivison S Piccirillo C Steiner TS Levings MK Human CD4+ FOXP3+ regulatory T cells produce CXCL8 and recruit neutrophils Eur J Immunol 2011 15 306 312 21268001 10.1002/eji.201040459

[B34] Stoop JN Robinson JH Hilkens CM Developing tolerogenic dendritic cell therapy for rheumatoid arthritis: what can we learn from mouse models? Ann Rheum Dis 2011 15 1526 1533 21804099 10.1136/ard.2011.151654

[B35] Haldorsen K Moen K Jacobsen H Jonsson R Brun JG Exocrine function in primary Sjogren syndrome: natural course and prognostic factors Ann Rheum Dis 2008 15 949 954 17962240 10.1136/ard.2007.074203

[B36] Jonsson MV Hammenfors D Brun JG Jonsson R Ultrasonography of major salivary glands in primary Sjogren's syndrome Arthritis Rheum 2012 15 S925 25535773

[B37] Zou Q Jiao J Zou MH Xu JH Pan YF Chen JN Cheng MH Zhang F Zhang Y Semi-quantitative evaluation of salivary gland function in Sjogren's syndrome using salivary gland scintigraphy Clin Rheumatol 2012 15 1699 1705 22941258 10.1007/s10067-012-2076-3

