# Supplementary figures and images for: In vitro suppression of immune responses using monocyte-derived tolerogenic dendritic cells from patients with primary Sjögren's syndrome
Source: Arthritis Res Ther. 2013 Sep 9;15(5):R114. doi: 10.1186/ar4294 (PMC3978468; doi:10.1186/ar4294)

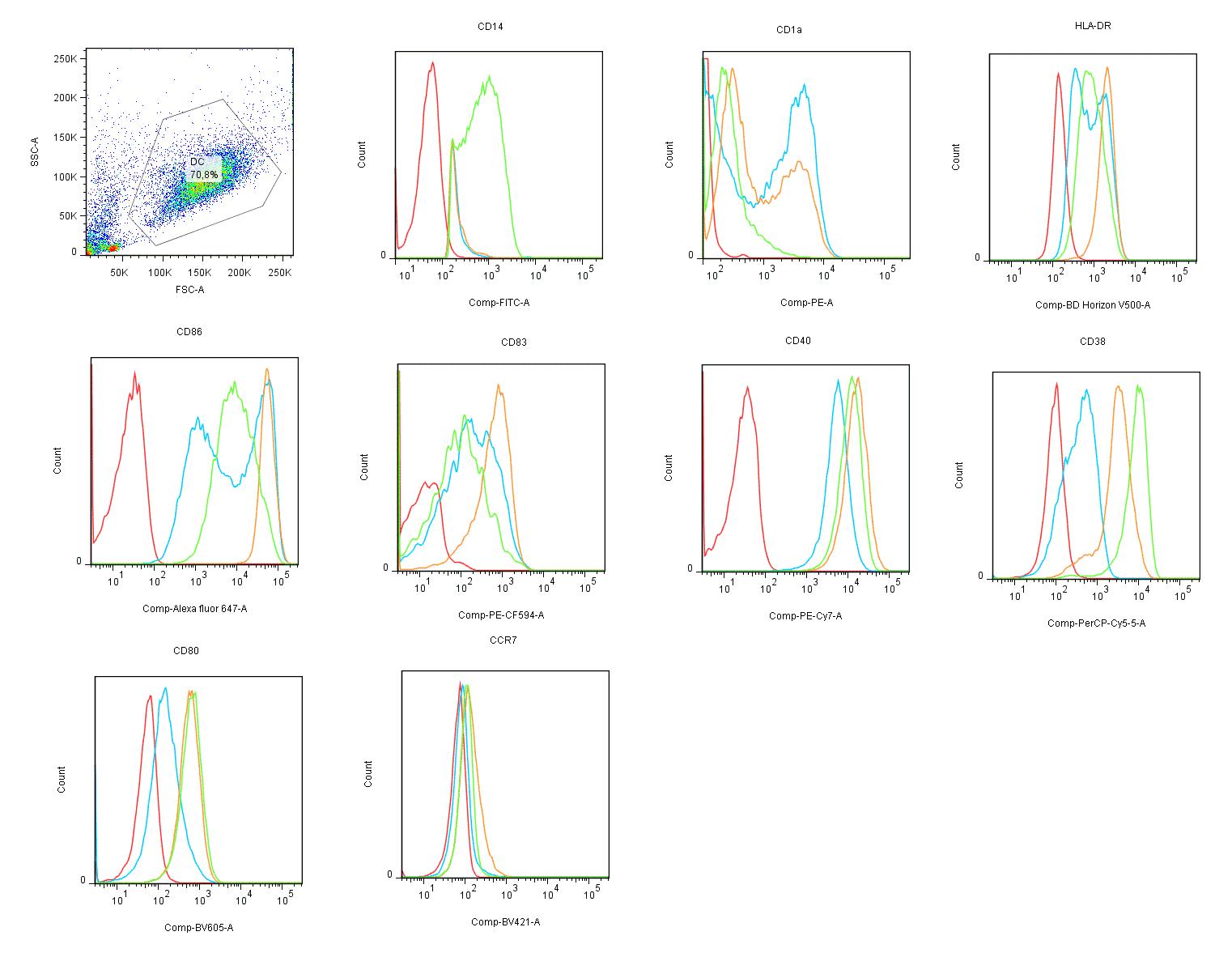

Supplement: Additional file 1 — Figure S1. DexVD3 DC have a semi-mature macrophage-like phenotype. The DC were gated based on forward and side scatter dot plots and the expression of surface markers was analyzed. Each histogram shows the negative control (red), immature DMSO DC (blue), mature DMSO DC (orange) and DexVD3 DC (green). [file ar4294-S1.JPEG]

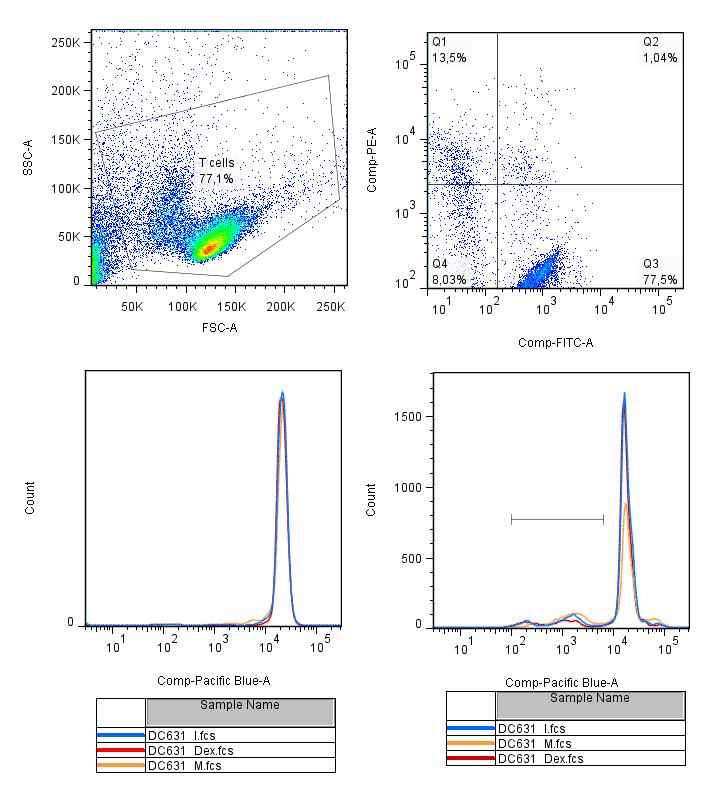

Supplement: Additional file 2 — Figure S2. Gating strategy for the analysis of T cell proliferation after priming with various DC groups. The top left dot plot depicts cells in a live gate based on forward scatter and side scatter, the top right dot plot depicts gating on CD4+ and CD8+ T cells. The bottom left histogram represents proliferation of CD8+ T cells and the bottom right histogram shows the proliferation of CD4+ T cells. [file ar4294-S2.JPEG]

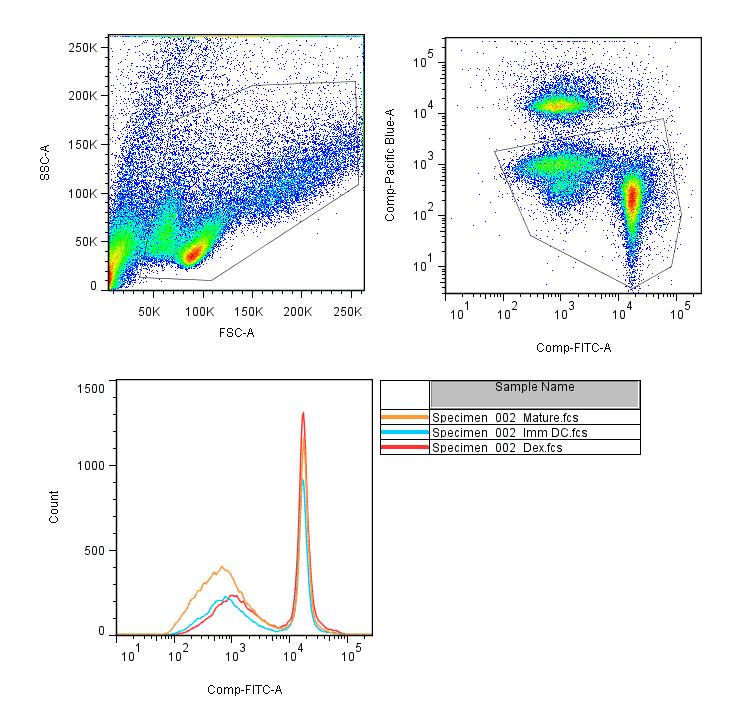

Supplement: Additional file 3 — Figure S3. Gating strategy for the analysis of the suppressive capacity of lymphocytes primed with various tolDC populations. Naïve responder cells were labeled with CFSE and tolDC-primed NAC were labeled with CellTrace Violet. This allowed exclusive gating on CellTrace Violet-negative cells and further analysis of proliferation of CFSE-labeled responder cells. The top left dot plot depicts cells in a live gate based on forward scatter and side scatter, the top right histogram depicts gating on CellTrace Violet-negative cells. The bottom histogram shows proliferation of CFSE-labeled CellTrace Violet-negative responder cells after co-culture with differently primed NAC. MFI values of proliferated responder cells and tolDC populations that were used to stimulate effector cells are shown in the histograms legend. MFI, median fluorescence intensity; NAC, nonadherent cells; tolDC, tolerogenic dendritic cells. [file ar4294-S3.JPEG]
